# A genome-wide search for eigenetically regulated genes in zebra finch using MethylCap-seq and RNA-seq

**DOI:** 10.1038/srep20957

**Published:** 2016-02-11

**Authors:** Sandra Steyaert, Jolien Diddens, Jeroen Galle, Ellen De Meester, Sarah De Keulenaer, Antje Bakker, Nina Sohnius-Wilhelmi, Carolina Frankl-Vilches, Annemie Van der Linden, Wim Van Criekinge, Wim Vanden Berghe, Tim De Meyer

**Affiliations:** 1 Department of Mathematical Modelling, Statistics and Bioinformatics, University of Ghent, Ghent, Belgium; 2 Laboratory of Protein Chemistry, Proteomics and Epigenetic Signalling (PPES), Department of Biomedical Sciences, University of Antwerp, Antwerp, Belgium; 3 Department of Behavioral Neurobiology, Max Planck Institute for Ornithology, Seewiesen, Germany; 4 Bio-Imaging Lab, Department of Biomedical Sciences, University of Antwerp, Antwerp, Belgium

## Abstract

Learning and memory formation are known to require dynamic CpG (de)methylation and gene expression changes. Here, we aimed at establishing a genome-wide DNA methylation map of the zebra finch genome, a model organism in neuroscience, as well as identifying putatively epigenetically regulated genes. RNA- and MethylCap-seq experiments were performed on two zebra finch cell lines in presence or absence of 5-aza-2′-deoxycytidine induced demethylation. First, the MethylCap-seq methodology was validated in zebra finch by comparison with RRBS-generated data. To assess the influence of (variable) methylation on gene expression, RNA-seq experiments were performed as well. Comparison of RNA-seq and MethylCap-seq results showed that at least 357 of the 3,457 AZA-upregulated genes are putatively regulated by methylation in the promoter region, for which a pathway analysis showed remarkable enrichment for neurological networks. A subset of genes was validated using Exon Arrays, quantitative RT-PCR and CpG pyrosequencing on bisulfite-treated samples. To our knowledge, this study provides the first genome-wide DNA methylation map of the zebra finch genome as well as a comprehensive set of genes of which transcription is under putative methylation control.

In the past few decades, songbirds became a widely used model system in the behavioral neurobiology of learning^1^, evolutionary genetics^2^, neuroendocrinology^3^ – particularly the sexual differentiation of the brain – and adult neurogenesis^4^. Songbirds evolved the ability to learn vocalizations by copying a singing adult. Vocal learning is a trait that songbirds share with humans, where it forms the basis of spoken language acquisition, but which is absent in traditional model organisms such as rodents and nonhuman primates^5^. The presence of multiple behavioral parallels between song learning and human speech learning makes it plausible that there are similar underlying neurobiological pathways involved^6^,^7^. Indeed, it has become apparent that there are striking homologies between the brains of birds and mammals, leading to the identification of common neuronal and molecular substrates^8^.

One songbird in particular, the zebra finch (*Taeniopygia guttata*), has been the focus of many studies because of its rapid maturation and its tendency to sing and breed in captivity^5^,^7^, highlighted by the availability of its complete genome sequence^9^. Although numerous genomic resources are becoming available for this animal model, several *in vivo* experiments (e.g. knockdowns or manipulation of gene expression), are still impossible or very laborious and expensive. Fortunately, recently two immortalized zebra finch cell lines were established, i.e. the diploid male G266 and tetraploid female ZFTMA cell line, both obtained from spontaneous, non-neuronal tumors, providing an efficient alternative to whole-animal manipulations^10^. Although these cell lines are not derived from brain tissue, it has previously been observed that they express many neurobiologically relevant genes^11^.

Epigenetic modifications, including DNA methylation, have been shown to play an important role in several neurobiological and cognitive processes, such as learning and memory^12^,^13^. Epigenetics is defined as the study of inheritable chromatin modifications that have an impact on gene expression without altering the underlying DNA sequence^14^. DNA methylation is a well-known epigenetic mark that is essential for normal development. It is established by DNA methyltransferases (DNMTs) and involves the transfer of a methyl group to cytosine residues at the carbon 5 position, predominantly in a CpG context. This methylation mark is known to regulate gene expression and when located in the promoter region, it generally leads to transcriptional silencing of the corresponding gene^15^. DNA methylation has traditionally been regarded as a highly stable epigenetic mark in post-mitotic cells, defining their cellular identity. However, it has been observed that the postnatal brain shows stimulus-induced *de novo* CpG methylation or active DNA demethylation at specific loci during the process of learning and memory formation^16^,^17^,^18^. For example, there is accumulating evidence that the expression of brain-derived neurotrophic factor (*BDNF*) is regulated by epigenetic modifications, and in particularly by promoter DNA methylation^19^. BDNF is a neurotrophic factor important for neuronal activity-dependent processes such as long term-potentiation, neuronal survival, development, memory formation and synaptic plasticity, which is linked to several neurological disorders e.g. schizophrenia and mood disorders^20^,^21^.

Even though zebra finches are an attractive model to study the molecular basis of (vocal) learning, a genome-wide DNA methylation pattern in this species is currently lacking. In order to assess a first ‘draft of the zebra finch methylome’, we performed methyl-CpG binding domain protein sequencing (MethylCap-seq) experiments on the G266 as well as the ZFTMA cell line, in presence or absence of the DNA methylation inhibitor 5-aza-2′-deoxycytidine (AZA) that, when incorporated in the genome, inhibits DNMTs resulting in a net demethylation effect^22^. MethylCap-seq is based on the specific enrichment of methylated portions of the genome (i.e. enrichment-based method) by Methyl-CpG Binding Domain (MBD)-based affinity purification followed by massively parallel sequencing^23^,^24^. Contrasting whole-genome bisulfite sequencing, it provides a cost-efficient option to assess genome-wide DNA methylation profiles^25^. However, until now MethylCap-seq has primarily been used for human and mouse genomes, and its effectiveness has not yet been demonstrated in zebra finch. Here, we evaluate the quality and sensitivity of this methodology for zebra finch by comparison with a species-independent, though not genome-wide, methodology, i.e. Reduced Representation Bisulfite Sequencing (RRBS)^26^. By comparing MethylCap-seq data of dimethyl sulfoxide (DMSO, solvent control) and AZA-treated samples it was possible to identify genomic regions that are substantially demethylated due to the treatment. Moreover, to assess the effect of AZA-induced demethylation on gene expression RNA-seq experiments were performed, which were validated by performing zebra finch Exon Array analysis. Subsequently, a pathway analysis was accomplished on the resulting set of loci, and a subset was validated by independent methodologies i.e. CpG pyrosequencing and quantitative Real-Time PCR (qPCR).

To our knowledge, this study provides the first genome-wide draft methylome of the zebra finch. The inclusion of AZA-treated samples allows the identification of loci, which are nearly always methylated, or – more importantly – for which the methylation status is highly variable in different settings. We provide an overview of loci featured by dynamic methylation degrees as well as a list of genes for which expression is putatively regulated by DNA methylation in the promoter region.

## Materials and Methods

### Cell cultures and treatments

Two zebra finch cell lines were used: the diploid male G266 cell line and the tetraploid female ZFTMA cell line, both derived from spontaneous tumors^10^. These cell lines were a kind gift of Dr. David Clayton (Queen Mary University of London). Cells were cultured in DMEM high glucose medium (Life Technologies 41965039) supplemented with 10% heat inactivated fetal bovine serum (Life Technologies 102070106), 2% heat inactivated chicken serum (Life Technologies 15140122) and 1% penicillin streptomycin (Life Technologies 15070063). Cultures were maintained at 37 °C, 5% CO_2_ and 95–98% humidity.

Cells were treated for 72 hours with 1 μM concentration of AZA (=demethylation treatment, Sigma-Aldrich A3656), with DMSO as a solvent control, or left untreated. Both AZA and DMSO inductions were repeated every 24 hours, as AZA is known to be relative unstable in aqueous solution. Genomic DNA (gDNA) was extracted from an untreated ZFTMA cell line as well as from AZA- and DMSO-treated G266 and ZFTMA cell lines using the DNeasy mini kit (Qiagen 69506) while RNA was extracted from AZA- and DMSO-treated G266 and ZFTMA cell lines using Trizol and RNeasy mini kit (Qiagen 74106), both according to the manufacturer’s instructions. DNA and RNA concentrations were measured using Nanodrop 1000 (Thermo Fisher Scientific).

### Library preparation and sequencing

#### Reduced Representation Bisulfite Sequencing

Bisulfite treatment, library preparation and sequencing of the untreated ZFTMA DNA sample were performed as a commercial service by BaseClear, using the Zymo Research EpiQuest bisulfite and library preparation procedures. For this sample, 500 ng gDNA was checked for integrity and lack of degradation by agarose gel electrophoresis and the concentration was measured by a non-UV method. The DNA was subsequently fragmented, size selected (150–450 bp) and a bisulfite conversion was performed. Next, Illumina adapters were added and the fragments amplified with a PCR amplification. The length distribution of the resulting library was checked on a BioAnalyzer (Agilent Technologies G2940CA) and quantified. Quantified and converted DNA libraries were loaded on the Illumina HiSeq2000 platform and a paired-end sequencing run was performed as subscribed in the Illumina protocol ‘performing a multiplexed paired-end run’ (2 times 50 cycles).

#### Methyl-CpG binding domain protein sequencing

Methyl-CpG binding domain protein sequencing (MethylCap-seq), which combines enrichment of methylated DNA fragments by MBD-based affinity purification with massively parallel sequencing^23^, was used to profile the DNA methylation pattern of the DMSO-, AZA- and untreated zebra finch cell lines, i.e. G266 and ZFTMA. The gDNA was sheared using Covaris S2 equipment with following settings: duty cycle 10%, intensity 5, 200 cycles per burst during 190 seconds to obtain fragments with an envisaged average length of 200 bp. The power mode was frequency sweeping, temperature 6–8 °C and water level of 12. 2 μg was loaded in 130 μl TE (1:5) in a microtube with AFA intensifier. After DNA fragmentation, the methylated fragments were captured using Diagenode’s MethylCap^TM^ kit (Diagenode AF-100-0048) according to the manufacturer’s instructions starting from a DNA concentration between 250 and 500 ng. Captured DNA was eluted in a 150 μl High Elution buffer and purified with MinElute PCR purification columns (Qiagen 28006). Purified DNA was used for library preparation, which was performed on the Apollo 324^TM^ (IntegenX) using the Multiplexing Sample Preparation Oligonucleotide Kit (Illumina PE-400-1001). Size selection (100–350 bp) was done with Agencourt AMPure XP beads (Beckman Coulter Inc. (Analis SA) A63882) in combination with polyethylene glycol (PEG). 22 μl of DNA was subjected to PCR following the Illumina Library Amplification Index Protocol (Illumina) with 21 cycles of PCR amplification using Phusion High-Fidelity PCR Master Mix (New England BioLabs (NEB) M0532L) with primers of the Multiplexing Sample Preparation Oligonucleotide Kit (Illumina). PCR products were purified with the High Pure PCR Cleanup Micro Kit (Roche Applied Science 04983912001). Next, libraries were assessed using an Agilent DNA 1000 Chip (Agilent Technologies 5067–1504). The concentration was determined following Illumina’s qPCR Quantification Protocol Guide (Illumina SY-930-1010). After pooling the libraries, paired-end sequencing was performed as a commercial service by BaseClear on one lane (8 samples/lane) of the Illumina HiSeq2000 following the Illumina protocol ‘performing a multiplexed paired-end run’ (2 times 50 cycles).

#### RNA-sequencing

RNA-seq experiments were performed on DMSO- and AZA-treated G266 and ZFTMA cell lines. RNA samples were analyzed with both the Quant-iT^TM^ Ribogreen^**^®^**^ RNA Assay Kit (Invitrogen R11491) and the Agilent Eukaryote Total RNA 6000 Pico Chip (Agilent 5067–1513) to ensure adequate quality and quantity of total RNA. Prior to the library preparation, an rRNA depletion step was performed on 3 μg input RNA (Ribogreen^**^®^**^ measurement) using the Ribo-Zero^TM^ Magnetic Kit (Human/Mouse/Rat, Epicentre MRZH11124). Next, cDNA libraries for the ZFTMA and G266 rRNA depleted samples (6 μl/sample) were prepared using the ScriptSeq^TM^ v2 RNA-seq Library Preparation Kit (Epicentre SSV21124) following the manufacturer’s protocol. Library amplification was performed with 15 PCR cycles. Quality control of the libraries was assessed using an Agilent High Sensitivity DNA Chip (Agilent Technologies 5067–4526). The concentration was determined following Illumina’s qPCR Quantification Protocol Guide (Illumina SY-930-1010). After pooling the libraries, paired-end sequencing was again performed by BaseClear on one lane (4 samples/lane) of the Illumina HiSeq2000 again following Illumina’s protocol ‘performing a multiplexed paired-end run’ (2 times 50 cycles).

### Sequence read mapping

For each sample, MethylCap-seq paired-end reads were mapped with Bowtie2 (v2.1.0)^27^. The mapping parameters were chosen so that only paired-end reads that mapped uniquely and concordantly on the zebra finch reference genome (assembly taeGut3.2.4, Ensembl release 72) within a maximum of 400 bp of each other were retained. Seed mismatches, length and interval during multiseed alignment were set to the default values. For RRBS, paired-end reads were mapped with Bismark (v0.9.0) in Bowtie2-mode^28^. Again, default values were chosen for the multiseed alignment and only concordantly, uniquely mapped reads with a maximum insert size of 500 bp were withheld. Paired-end RNA-seq reads were mapped with STAR (v2.3.0)^29^. Here, reads mapping up to 10 places on the zebra finch genome were allowed with a maximum of 6 mismatches per fragment, i.e. read pair. For MethylCap-seq, duplicate fragments, i.e. fragments with the exact same location of both paired-end reads, were disposed as these are most likely the result of amplification of the same sequence reads during library preparation. As for RNA-seq and RRBS duplicate reads are expected – they arise from both the enzymatic cutting (RRBS)/limited library complexity (RNA-seq) as well as from the PCR step and there is no simple way to distinguish between these two sources of duplicate reads – they were retained for further analysis.

### Quality control of MethylCap-seq

As our study is the first to use MethylCap-seq – which employs a human-derived MBD – on zebra finch samples, it was necessary to first assess the accuracy of this methodology in this non-mammal species. MethylCap-seq specifically enriches for methylated genomic regions. Hence, a higher amount of MethylCap-seq fragments covering a particular region reflects in general a higher methylation degree. Though limited regarding genome-wide information, RRBS is based on bisulfite treatment – and therefore species-independent – and can yield a quantitative estimate of the percentage of methylation. As bisulfite treatment only converts non-methylated cytosine residues to uracil, which will be read as a thymine when sequenced, whereas methylated cytosine residues remain identified as cytosine, it is possible to summarize the methylation state of each sequenced cytosine and CG dinucleotide (=CpG methylation degree). In order to evaluate the efficacy of the performed MethylCap-seq in zebra finch, the MethylCap-seq data of the untreated ZFTMA cell line was compared to its corresponding RRBS data. For each RRBS-covered CpG (at least 10 RRBS reads), the CpG methylation degree was determined as well as its coverage obtained by MethylCap-seq. Finally, to indicate the sensitivity of MethylCap-seq it was examined if higher RRBS CpG methylation degrees are indeed associated with more MethylCap-seq fragments.

### Read summarization

Prior to the differential expression/methylation analysis, the sequencing reads needed to be assigned to genomic features of interest. The mapped RNA-seq reads were converted to fragment counts per exon and grouped per gene with the R-package Rsubread (v1.12.0)^30^. Gene annotations were obtained from Ensembl (release 72).

In order to summarize the MethylCap-seq reads, it was necessary to first identify all possibly methylated regions in the zebra finch genome (Methylation Peaks), which could subsequently be used as units for statistical analysis, cf. the use of genes in RNA-seq data analysis. As MethylCap-seq data consists of a mixture of DNA methylation signal and (relatively low amounts of) noise, only loci that exhibit a sufficiently high signal intensity (i.e. pass a certain threshold) qualify as “potentially methylated”. This classification was done by taking the summed coverage of both ZFTMA and G266 cell line control sample results (i.e. DMSO-treated), and by evaluating different coverage thresholds – ranging from 1 to 20 – for “significant” methylation. Instead of imposing an arbitrary signal intensity threshold, an optimal threshold was identified by using the RRBS data as “gold standard”. Consequently, for threshold identification, only those loci for which RRBS data were available were considered, but we made the reasonable assumption that the identified threshold can be applied on the complete MethylCap-seq dataset.

In a first step, the MethylCap-seq data for both the ZFTMA and G266 DMSO-treated samples was piled-up for an unbiased selection, hereby making the common assumption that the large majority of methylation events are conserved between both cell lines. Second, for each CpG measured by RRBS and MethylCap-seq coverage threshold under study, it was assessed whether this CpG was categorized as “methylated” (=MethylCap-seq intensity above threshold), or “unmethylated” (=vice versa). Third, the optimal coverage threshold was determined by comparing the RRBS methylation degrees between both categories using the Kruskal-Wallis rank sum test. Subsequently, the Methylcap-seq threshold for which this comparison yielded the clearest difference (i.e. lowest p-value) was selected as optimal threshold. In other words, for this threshold MethylCap-seq results correspond best with the RRBS results.

In a next step, the Methylation Peaks were defined as all genomic regions where every position has a total coverage larger than or equal to this optimal coverage threshold. Additionally, these regions were trimmed at both ends so that the final regions start and end with a CpG. Finally, for each sample, the non-duplicate, uniquely mapped MethylCap-seq reads could be summarized into fragment counts per Methylation Peak.

The corresponding functional genomic annotation (i.e. promoter, exon, intron and intergenic) of the identified Methylation Peaks was determined using Ensembl (release 72), wherein the promoter was defined as starting from 2000 bp upstream until 500 bp downstream of the transcriptional start site. Only if a Methylation Peak had none of the genic categories (i.e. promoter, exon, and intron) it was defined as intergenic.

Both circular plots (Circos tool^31^) and an in-house developed genome browser (H2G2, http://h2g2.ugent.be/biobix.html) were used for visualization of the experimental data.

### Differential methylation and expression analysis

To test whether the methylation pattern in intergenic regions was different between gene-deserts, i.e. gene-poor/intergenic regions >500 kb, and no-gene-deserts, i.e. intergenic regions <= 500 kb, the amount of reads in the Methylation Peaks falling in each group was determined. Per chromosome, the read count of each group was normalized for its respective length, after which a t-test was performed comparing the methylation level of both gene-deserts and no-gene-deserts.

Subsequently, we used the R-package EdgeR (v3.4.2)^32^ to test for both differential methylation degrees as well as differential gene expression between the DMSO- and AZA-treated samples. Normalization of the MethylCap-seq counts between the two treatments was performed based on the reads that mapped in non-Methylation Peaks, i.e. the library size of the “noise reads”. The rationale of this in-house method was that despite the performed treatment, the observed noise, i.e. amount of reads between Methylation Peaks, should be similar between the two G266 samples as well as between the two ZFTMA samples. Since the putative noise regions might still contain some signal as well, this can be considered a conservative approach. Normalization of the RNA-seq gene counts was performed with the PoissonSeq package implemented in R (v1.1.2)^33^. This normalization method is based on a Chi-square-like goodness-of-fit statistic and has been shown to perform better than other existing methods (e.g. trimmed mean of M-values (TMM)^34^, DESeq^35^, quantile and library size normalization). Because the global differences between the DMSO- (=control) and AZA-treated (=demethylation treatment) samples are of primary interest, and not the differences between the two cell lines nor the treatment effect that occurs for only one cell line, a paired design was chosen – which also accounts for the different ploidy levels between the two cell lines. Resulting p-values were adjusted using the Benjamini-Hochberg correction and only Methylation Peaks (or genes) that were significant at a false discovery rate (FDR) of 0.1 were considered as differentially methylated (or expressed).

### Enrichment analysis

Next, we tested the list of differentially methylated genomic regions between DMSO- and AZA-treated samples for enrichment in one or more functional categories (i.e. promoter, exon, intron and intergenic). By random sampling from the total amount of Methylation Peaks and counting the occurrences of the respective annotations, a null distribution was generated. During this sampling procedure, the number of Methylation Peaks sampled for each chromosome was equal to the number of significantly down-methylated Methylation Peaks for that chromosome. Sampling was repeated 1,000 times. Based on the null distribution obtained for promoter, exonic, intronic and intergenic regions it was possible to calculate a two-sided p-value for each of these categories.

For Methylation Peaks that were featured by more than one genic annotation (i.e. overlapping genes and/or different transcripts and/or sense and antisense strand and/or peaks overlapping different locations on same strand) the attributed score of the functional location was divided by the amount of different categories for this peak (the sum always being one). For example, if a Methylation Peak is located in an exon on the sense strand but is also located in an intron on the other strand, both “exon” and “intron” categories were attributed a score of 0.5.

As the AZA-treatment normally leads to demethylation and we were particularly interested in demethylation that results in upregulated expression, a similar analysis was performed only one those genomic regions which were significantly down-methylated and located in genes significantly upregulated by AZA-treatment. The functional genic annotation (i.e. promoter, exon and intron) of these Methylation Peaks was determined as described above. Likewise, two-sided p-values were calculated for each genic annotation, after random sampling from the whole set of Methylation Peaks located in upregulated genes.

### Ingenuity Pathway Analysis

To interpret the results in the context of biological processes, regulatory networks and other pathways, Ingenuity Pathway Analysis (IPA) (Ingenuity Systems^**^®^**^, www.ingenuity.com) was performed. The IPA software provides a comprehensive database of known networks and pathways that are constantly being updated based on published literature on gene functions and interactions. After providing the Ensembl gene identifiers of the target genes to IPA (fall release 2015) the most affected/involved biological processes and networks are listed and scored based on significance. In addition, also possible relationships with diseases and disorders are shown. The provided gene list consisted of genes that upon AZA-treatment were significantly upregulated and featured by at least one Methylation Peak exhibiting significant AZA-induced demethylation in the promoter region (i.e. genes that are under putative DNA methylation control). P-values were corrected for multiple testing using the Benjamini-Hochberg method.

### Validation: Zebra finch Exon Arrays and Quantitative Real Time-PCR

To confirm the RNA-seq findings, we performed zebra finch Exon Arrays (Affymetrix custom designed) on independent samples. Total RNA was extracted using Trizol and the RNeasy Mini Kit (#74104, Qiagen Inc., Valencia, CA, USA) according to the manufacturer’s protocol including the optional DNA digestion step. RNA quality was assessed using an Agilent Model 2100 Bioanalyzer (Agilent Technologies, Palo Alto, CA, USA) and the RNA concentration was determined with a Nanodrop 1000 spectrometer (Thermo Fisher Scientific, Wilmington, MA, USA). For each sample, 100 ng of total RNA was processed for hybridization on the microarray using the Ambion^®^ WT Expression Kit and (#4411974, Thermo Fisher Scientific Inc.), the GeneChip^®^ WT Terminal Labeling and Controls Kit (#901524, Affymetrix^®^ Microarray Solutions) and the GeneChip^®^ Hybridization, Wash, and Stain Kit (#900720, Affymetrix^®^ Microarray Solutions). The resulting cDNA was hybridized to the custom designed Affymetrix Gene Chip^®^ MPIO-ZF1s520811 Exon Array as described by Frankl-Vilches *et al*. 2015^36^ and scanned with the GeneChip^®^ Scanner 3000 7G (#00–0210, Affymetrix^®^ Microarray Solutions). CEL files generated by the Affymetrix^®^ GeneChip^®^ Command Console^®^ Software (AGCC) were imported into ChipInspector software, version 21 (El Dorado Database version: E30R1410 Genomatix GmbH). Differential expression between DMSO and AZA (500 nM) treated samples was analyzed using the group-wise exhaustive analysis with false discovery rate set to zero and 10-significant probe minimum coverage. CEL files, raw data and additional statistical information have been deposited in NCBI’s Gene Expression Omnibus^65^ and are accessible through GEO Series accession number GSE71344).

In order to determine whether the expression level correlates to the methylation status, 7 genes were selected that showed a significant increase in gene expression (both RNA-seq and Exon Arrays) and decrease in promoter DNA methylation (MethylCap-seq): *BDNF*^37^, neuroglobin (*NGB*)^38^, *HES* family BHLH transcription factor 1–4 (*HES1-4*)^39^, GABA(A) receptor subunit delta (*GABRD*)^40^, ankyrin 1 (*ANK1*)^41^, matrix metallopeptidase 9 (*MMP9*)^42^ and inhibitor of kappa light polypeptide gene enhancer in B-cells kinase epsilon (*IKBKE*). Three independent biological replicates of DMSO- and AZA-treated samples were used for the validation experiments. Gene-specific qPCR primers were designed using PrimerBlast (NCBI) (Table 1). Total RNA was isolated as described above. 1 μg of RNA was subsequently converted into cDNA using oligodT primers and M-MLV Reverse transcriptase (Promega). SYBR Green qPCR was performed in triplicate on the Rotor-GeneQ instrument (Qiagen) using the Rotor-Gene SYBR Green Fast PCR kit, as follows: 95 °C for 5 min, followed by 45 cycles of 95 °C for 5 s and 60 °C for 10 s. Dissociation curves were checked to ensure amplification of a single PCR product. Moreover, standard curves were run to check the amplification efficiency of each assay. All amplification efficiencies were between 100% and 111%, with no major differences between test genes and housekeeping genes.

For each independent biological sample, all cycle threshold (Ct) values for the target genes were normalized to the corresponding geometric mean of three housekeeping genes: ribosomal protein L30 (*RPL30*), ribosomal protein S13 (*RPS13*), and glyceraldehyde-3-phosphate dehydrogenase (*GAPDH*). To compare the expression levels between different treatments, the 2 ^−**ΔΔ**Ct^ method^43^ was used and corresponding gene-specific p-values were calculated with a two-way ANOVA – using treatment and sample (=1, 2 or 3) as factors – on the normalized Ct values instead of the 2 ^−**ΔΔ**Ct^ values.

### Validation: Single locus specific DNA methylation quantification by CpG pyrosequencing

Additionally, the MethylCap-seq profiles of *BDNF, NGB, HES1-4, GABRD, ANK1, MMP9* and *IKBKE* were validated with CpG pyrosequencing of bisulfite-treated samples (same three independent samples used for qPCR validation). PCR amplification and sequencing primers were designed using the Pyromark Assay Design v2.0 software (Qiagen). The primers (Table 2) were designed at significant Methylation Peaks in the promoter region of the genes of interest. To ensure sufficient bisulfite conversion, a bisulfite conversion control was included in each assay.

First, 2 μg of gDNA was bisulfite converted using the EpiTect Fast Bisulfite Kit (Qiagen). 20 ng of this bisulfite converted DNA was then used as a PCR template (reverse primer was biotinylated), using the PyroMark PCR kit (Qiagen). Primer annealing temperatures were optimized: 56 °C for *BDNF, GABRD, ANK1* and *MMP9*, 58 °C for *HES1-4*, 59 °C for *IKBKE* and 60 °C for *NGB*. Next, 15 μl of the PCR product was used for CpG pyrosequencing on the PyroMark Q24 instrument (Qiagen). Finally, the results were analyzed using the Pyromark Q24 software. Methylation values of each cell line were summarized per CpG for both the DMSO- and AZA-treatment and gene-specific p-values, i.e. including all CpGs tested for the gene, were obtained using a two-way ANOVA with treatment and CpG as factors.

## Results

### Experimental design

**Figure 1:**
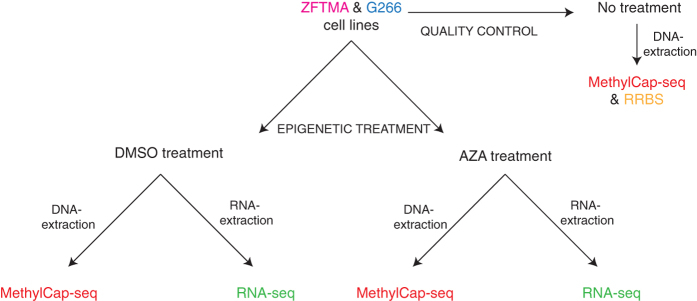
Overview of experimental design.

MethylCap-seq and RNA-seq analyses were performed on DMSO- and AZA-treated samples (Fig. 1). AZA is a chemical analogue of cytidine, that, when incorporated in the genome, inhibits DNMTs resulting in a net demethylation effect^22^. In addition, an untreated ZFTMA sample was analyzed with MethylCap-seq and RRBS to validate the MethylCap-seq methodology in zebra finch (quality control).

### Sequence read mapping

**Figure 2:**
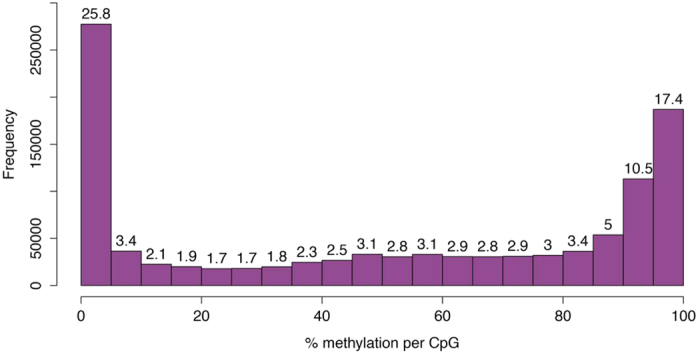
Histogram of %CpG methylation. CpG methylation degrees obtained with RRBS of untreated ZFTMA cell line (CpG sites with a RRBS coverage ≥10).

Table 3 lists the results of the sequence alignment for all libraries. For the five MethylCap-seq samples, removal of the duplicate fragments resulted in a lower fraction of non-duplicate, uniquely mapped reads for the AZA-treated experiments. Note that mapping of the RRBS paired-end reads resulted in a rather low percentage of uniquely mapped reads (28.43%), similar as Chatterjee *et al*.^44^ who obtained percentages ranging from 27% to 32.7% when uniquely mapping four non-mammal zebrafish RRBS libraries. In contrast, RRBS mapping of another non-model species (sheep) resulted in an average unique mapping efficiency of 60%^45^. These mapping efficiencies depend on both the specific characteristics of the reads and reference genome, as well as the used stringency during mapping (see Discussion). After RRBS alignment, the CpG dinucleotides were filtered based on coverage. Only CpG sites covered by 10 or more RRBS reads were retained for further analysis. For the RRBS library, this resulted in 1,074,648 CpGs (approximately 11% of total number of genomic CpG sites), with a mean coverage of 123 for which subsequently CpG methylation degrees were calculated (Fig. 2).

### MethylCap-seq is an appropriate methodology for studying DNA methylation in zebra finch

**Figure 3:**
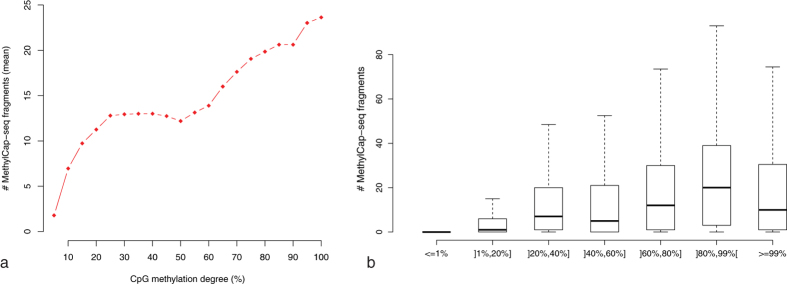
Comparison of MethylCap-seq and RRBS for the RRBS-covered CpGs (coverage ≥10). (**a**) Plot of the obtained RRBS CpG methylation degree and the average MethylCap-seq coverage. (**b**) Boxplots of the observed MethylCap-seq fragments for RRBS-covered CpGs with a CpG methylation degree starting from ≤1% to ≥99%, respectively.

Since this is the first study performing MethylCap-seq on zebra finch samples, prior to the genome-wide DNA methylation analysis, it was necessary to evaluate the effectiveness of this technique in zebra finch. Therefore, as a quality control, the untreated ZFTMA sample was also analyzed with RRBS, demonstrating that higher RRBS CpG methylation degrees are clearly associated with more MethylCap-seq fragments (Fig. 3): for very low RRBS methylation percentages almost no fragments are picked up (Fig. 3a,b), whereas for very high methylation percentages, on average a high coverage is obtained (Fig. 3a). Note that Fig. 3b also illustrates that even for ~100% methylated loci, no fragments may be picked up by MethylCap-seq, most likely due to several known biases inherent to the methodology^46^. Taken altogether, these results indicate that MethylCap-seq is an appropriate methodology for genome-wide mapping of DNA methylation in zebra finch, though only semi-quantitatively.

### (Differential) methylation and expression analysis

By combining the MethylCap-seq data with the RRBS data, we identified 719,917 possibly methylated genomic regions, i.e. ‘Methylation Peaks’, of which 60.29%, 31.32%, 5.39% and 3% are distributed in intergenic, intronic, exonic and promoter regions, respectively (see Supplementary Figure S1(a)). This peak distribution is not at all surprising, given the overall higher share of intronic/intergenic regions compared to exonic/promoter regions in the genome.

Examination of the methylation level per chromosome revealed overall similar methylation degrees between chromosomes (Supplementary Table S6). Furthermore, the distribution of methylation between exons, introns, promoters and intergenic regions showed only slight differences between chromosomes (Supplementary Figure S2). Additionally, it was examined if the intergenic methylation pattern distinguishes gene-deserts (gene-poor regions >500 kb) from other intergenic regions. Indeed, after determining the methylation level of each group per chromosome, a t-test showed that gene-deserts are clearly less methylated than other intergenic regions (p-value = 0.0079 for the 21 chromosomes characterized by gene-deserts) (data not shown).

In a next step, a quantitative comparison was made between the DMSO- (=control) and AZA-treated (=demethylation treatment) samples making use of the R-package EdgeR^32^. Normalization was based on the reads that mapped between Methylation Peaks. Using an FDR of 10% (corresponding with a p-value of 0.0043), 30,700 Methylation Peaks (=4.26%) showed significantly less methylation after AZA-treatment. 1,996 of the 18,318 annotated zebra finch genes (=10.90%) had at least one of these 30,700 Methylation Peaks in their promoter region (2000 bp up- and 500 bp downstream of transcription start site) (Supplementary Table S1).

To assess the impact of (variable) methylation on gene expression and to identify genes that are under DNA methylation control, RNA-seq experiments were performed. Of the mapped RNA-seq reads, only approximately 50% were located in genomic regions that are annotated by Ensembl as coding regions, i.e. exons, likely reflecting a still incomplete annotation of the zebra finch genome. For each sample, on average 70% of the known 18,318 genes were covered with at least 10 reads in their coding region. Next, data normalization was performed with PoissonSeq^33^ as this method performed better upon visual inspection – profile of the scaling factor line through the scatterplot of the data points – compared to other normalization methods (e.g. TMM^34^, quantile and library size normalization) (data not shown). After normalization and EdgeR analysis (FDR = 0.1, corresponding p-value = 0.0205), we identified 147 and 3,679 genes that were significantly down- and upregulated after AZA-treatment, respectively (Supplementary Tables S3 and S4).

**Figure 4:**
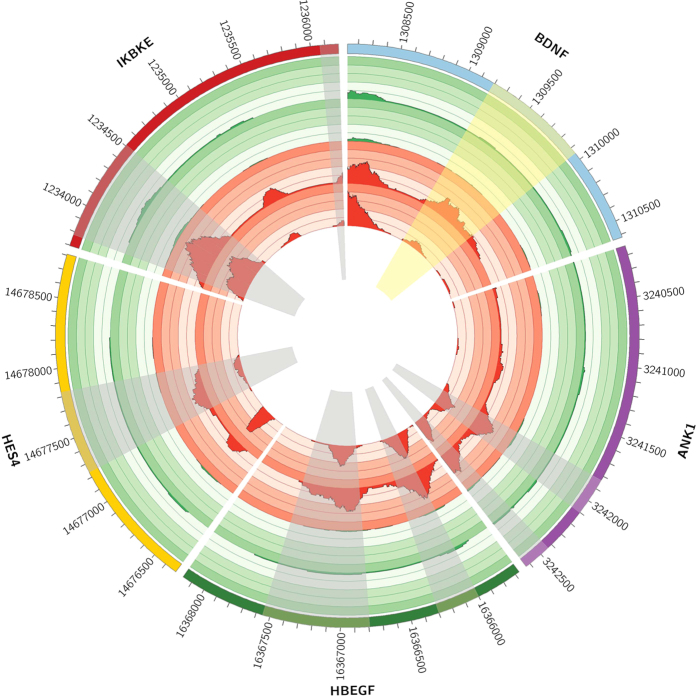
Circular representation of the normalized methylation profiles of 5 genes (*BDNF, ANK1, HBEGF, HES4* and *IKBKE)*. Promoter regions of the 5 depicted genes with the corresponding methylation profiles for both DMSO- and AZA-treatments. From inner to outer circle: (i, ii) MethylCap-seq data of DMSO-treated ZFTMA and G266 cell lines, respectively. (iii, iv) MethylCap-seq data of AZA-treated ZFTMA and G266 cell lines, respectively. (v) Gene promoter annotation. Significantly down-methylated Methylation Peaks are highlighted in grey. Apart from *BDNF*, all genes are part of the 357 genes found to be putatively regulated by promoter methylation (i.e. AZA-induced down-methylated promoter methylation and upregulated expression), while for *BDNF* only expression was significantly upregulated. For *BDNF,* the defined Methylation Peak in the promoter region for which methylation was clearly reduced – yet not significant at FDR 0.1 – is highlighted in yellow. From this figure it is clear that these genes are characterized by AZA-induced demethylation of the promoter region.

**Figure 5:**
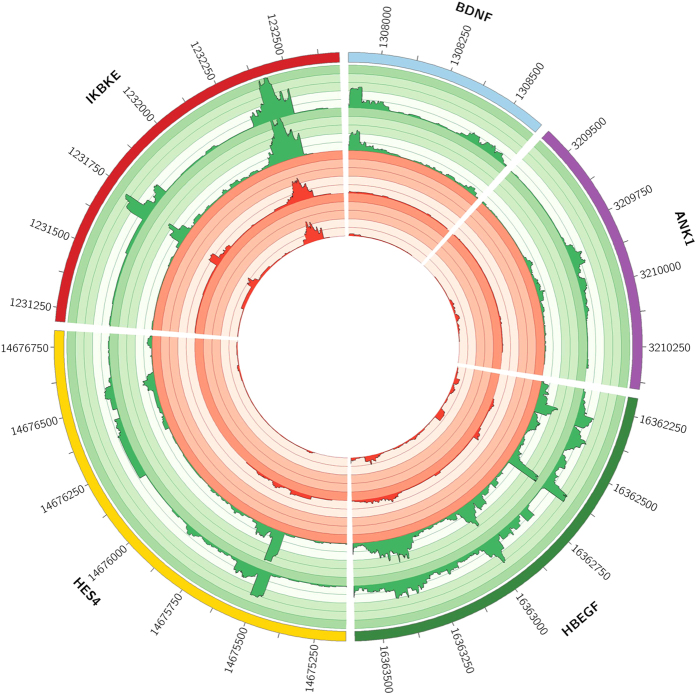
Circular representation of the normalized expression profiles of 5 genes (*BDNF, ANK1, HBEGF, HES4* and *IKBKE*). Genic regions with the corresponding expression profiles for both DMSO- and AZA-treatments. From inner to outer circle: (i, ii) RNA-seq data of DMSO-treated ZFTMA and G266 cell lines, respectively. (iii, iv) RNA-seq data of AZA-treated ZFTMA and G266 cell lines, respectively. (v) Genic annotation (Ensembl). Due to the large extent of some genes, instead of the whole genic range, a subregion of *HBEGF, ANK1* and *IKBKE* is shown. In both figures, the two G266 tracks are equally scaled, as well as both ZFTMA tracks. Apart from *BDNF*, all genes are part of the 357 genes found to be putatively regulated by promoter methylation (i.e. AZA-induced down-methylated promoter methylation and upregulated expression), while for *BDNF* only the expression was significantly upregulated. From this figure it is clear that these genes are characterized by AZA-induced re-expression.

Next, RNA- and MethylCap-seq results were compared. 357 of the 3,679 upregulated genes (=10.35%) were featured by at least one Methylation Peak exhibiting significant AZA-dependent demethylation in the promoter region, and are thus under putative DNA methylation control (Supplementary Table S2). As an example, the data tracks of a couple of these genes are depicted for both cell lines in two circular plots^31^. Figure 4 shows the methylation profile (=MethylCap-seq tracks) of the promoter regions of 4 genes that are putatively epigenetically regulated (i.e. *HES1-4,* heparin-binding EGF-like growth factor (*HBEGF*), *ANK1* and *IKBKE*), while Fig. 5 also shows the corresponding expression profile (=RNA-seq tracks) for these genes. As songbirds are an ideal model to study memory formation, brain development and neuroplasticity, two of the depicted genes are neurobiologically relevant (*HES1-4* and *ANK1)*, but in order to provide a general overview also two other genes (*HBEGF* and *IKBKE*) are added to the circular plots. These figures clearly show that these genes are characterized by AZA-induced demethylation of the promoter as well as re-expression.

In addition, also the MethylCap- and RNA-seq tracks of *BDNF* are shown. As stated earlier, it has been shown that *BDNF* is involved in synaptic plasticity with an expression inversely correlated with promoter DNA methylation. In our analysis, although *BDNF* expression was significantly upregulated after AZA-treatment (p-value = 5.31E-11, Fig. 5), we found no significantly demethylated Methylation Peaks in the *BDNF* promoter. However, visual inspection of the methylation profiles of the promoter region (Fig. 4) suggests considerable AZA-induced demethylation. Indeed, one particular Methylation Peak (highlighted in yellow in Fig. 4) received a p-value of 0.01 and despite the fact that this was just above the FDR threshold (corresponding with a p-value of 0.0043), this still suggests methylation-dependent gene expression.

### Enrichment analysis

**Figure 6:**
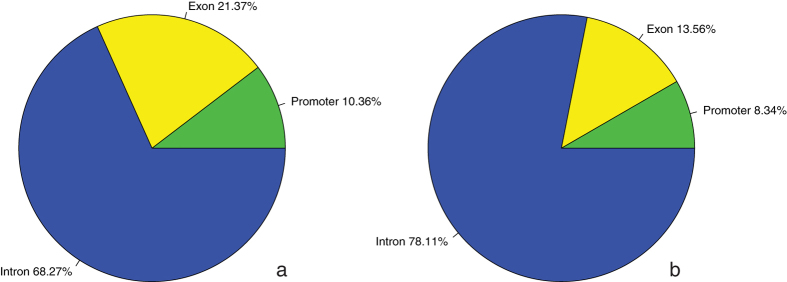
Genic distribution of the Methylation Peaks located in the upregulated genes after AZA-treatment. (**a**) Distribution of the Methylation Peaks featured by significant AZA-induced demethylation. (**b**) Mean genic classification of random Methylation Peaks resulting from 1,000 iterations.

As we aimed at the identification of epigenetically regulated genes, we were particularly interested in DNA demethylation leading to upregulated gene expression. Therefore, we determined the global distribution of the functional genic locations (i.e. promoter, exon and intron) of those AZA-demethylated Methylation Peaks that are located in genes upregulated after AZA-treatment (Fig. 6(a)). Not unexpectedly – given the higher share of intronic regions compared to exonic/promoter regions in the genome – the majority of these Methylation Peaks are located in intronic regions (68.27%). Additionally, a considerable number was found in the exonic (21.37%) and promoter regions (10.36%). In order to investigate whether one of these genic locations was relatively under- or overrepresented compared to random data, i.e. significantly decreased or enriched, we performed an enrichment analysis. By comparing the genomic distributions of 1,000 random samples (Fig. 6(b)) with the distribution shown in Fig. 6(a), a significant overrepresentation of AZA-demethylated loci was found for promoter and exonic regions whereas a significant underrepresentation was found for intronic regions (p-values < 0.001). No significant enrichment was found for a particular exon number (data not shown).

When looking only at the genome-wide methylation data, i.e. including intergenic regions and not taking into account the RNA-seq expression data, similar results were obtained: compared to random data, AZA-induced demethylation was relatively enriched in exonic and promoter regions, but decreased in intronic and intergenic regions (p-values < 0.001, see Supplementary Figure S1(b)).

### Ingenuity Pathway Analysis

Table 4 shows the top overrepresented disease and function categories resulting from an IPA of the 357 putatively epigenetically regulated genes (i.e. upregulated expression and down-methylated promoter after AZA-treatment). As can be noted from Table 4, neurological disease is classified as the top overrepresented disease category (Benjamini-Hochberg adjusted p-values between 6.1E-5 and 6.41E-2). Next to neurological diseases, this gene cluster also shows significant association with cancer and psychological disorders. A list of genes that contribute to each of the significant categories as well as a list of genes that contribute to each of the specific diseases or functions in each of the categories can be found in Supplementary Table S7 and Table S8, respectively.

### Validation: Zebra finch Exon Arrays, qPCR and single locus specific DNA methylation quantification by CpG pyrosequencing

In order to confirm the RNA-seq findings using a larger number of biological replicates, we performed zebra finch Exon Arrays (Affymetrix, custom designed). In addition, the inverse correlation between DNA methylation and expression levels was validated using pyrosequencing and qPCR for a subset of genes. 14 AZA-treated RNA samples, corresponding to 7 ZFTMA and 7 G266 samples, were compared to 11 controls (DMSO-treated), 5 from ZFTMA and 6 from G266 using Exon Arrays. With this platform, it is possible to detect expression of 17,882 Ensembl-NCBI annotated genes. 3,826 genes were found to be differentially expressed in the RNA-seq experiment, out of which 3,533 are present on the array. Hence, the validation covered 92.3% of the RNA-seq differentially expressed genes. Genes were considered differentially expressed when the p-value was lower than or equal to 0.05. This way, differential expression could be confirmed for 2,398 out of 3,826 (62.7%) genes: 109 out of 147 (74.1%) down-regulated genes and 2,289 out of 3,679 (62.2%) up-regulated genes.

In order to determine whether the expression level correlates to the methylation status, 7 genes were selected that showed a significant increase in gene expression and decrease in promoter DNA methylation in both RNA-seq and Exon Arrays and in MethylCap-seq experiments, respectively: *BDNF, NGB, HES1-4, GABRD, ANK1, MMP9* and *IKBKE*. To validate our findings three independent biological samples were treated with either DMSO or AZA and for these 7 genes (i) qPCR was carried out to validate the induction of mRNA expression and (ii) CpG pyrosequencing was performed to validate DNA methylation changes in the promoter region of the corresponding genes.

**Figure 7:**
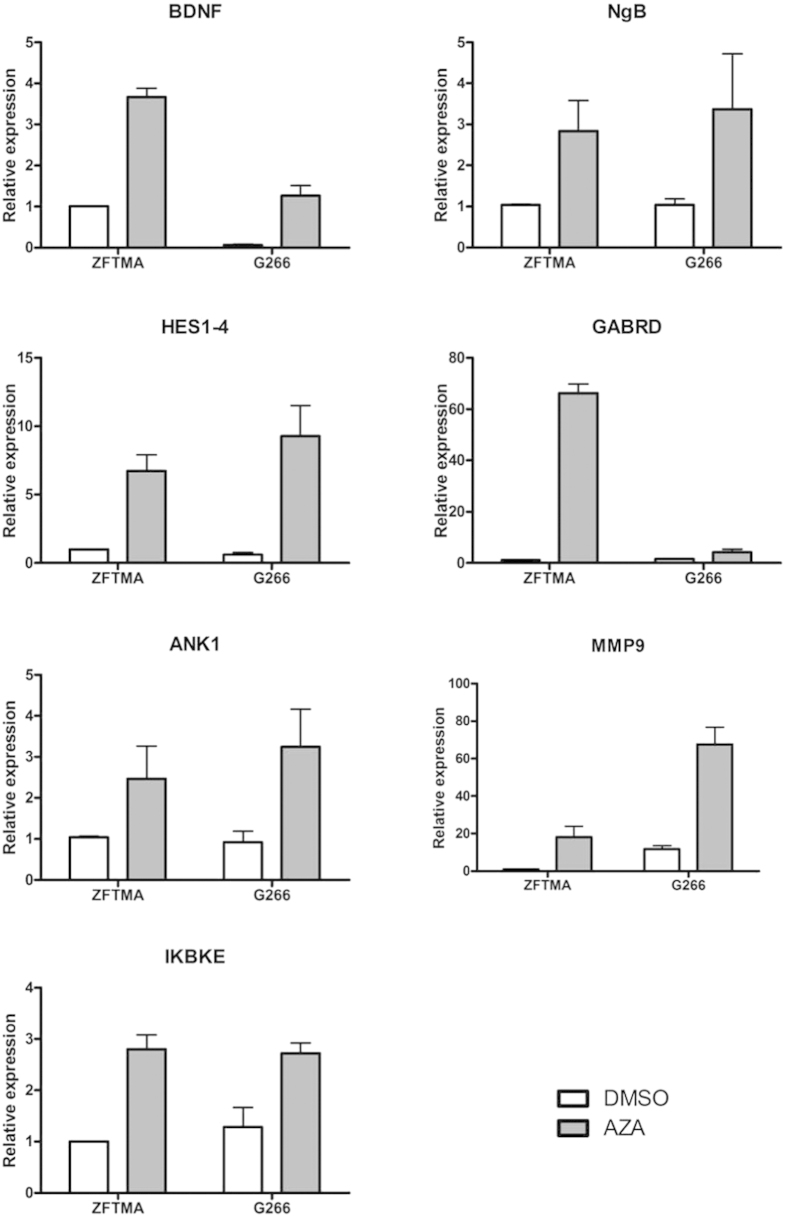
qPCR validation of RNA-seq results. For validation 7 genes were selected that showed a significant increase in gene expression and decrease in promoter DNA methylation in RNA-seq, Exon Arrays and MethylCap-seq experiments, respectively. This figure shows the impact of AZA (1 μM) treatment on *BDNF, NGB, HES1/4, GABRD, ANK1, MMP9* and *IKBKE* gene expression in ZFTMA and G266 cell lines, relative to a DMSO solvent control. Results were normalized to the geometric mean of reference genes *RPS13, RPL30* and *GAPDH*. The bar graphs represent relative mRNA expression (mean ± SEM) of three independent experiments. In all cases, the changes in mRNA expression after AZA treatment could be validated by qPCR (2-way-ANOVA).

**Figure 8:**
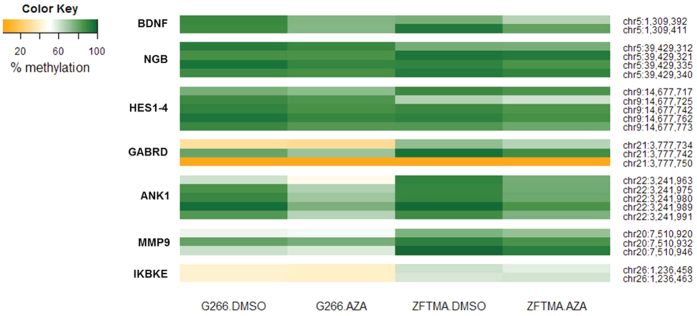
CpG pyrosequencing validation of MethylCap-seq results. For validation 7 genes were selected that showed a significant increase in gene expression and decrease in promoter DNA methylation in RNA-seq, Exon Arrays and MethylCap-seq experiments, respectively. This figure depicts changes in DNA methylation of specific CpG sites in the promoters of *BDNF, NGB, HES1/4, GABRD, ANK1, MMP9* and *IKBKE* after AZA (1 μM) treatment in ZFTMA and G266 cell lines, relative to DMSO solvent control. The shown DNA methylation levels represent the mean of three independent experiments. Only CpG sites that passed quality control were considered. The *NGB* gene was found to contain a SNP in the G266 cell line at position chr5:39,429,316, which was corrected for in the analysis. Methylation changes could be validated for each of the genes, except for *IKBKE* (2-way ANOVA). For *MMP9* the change in promoter methylation was only confirmed in the ZFTMA cell line.

For each gene, qPCR results were consistent with the obtained RNA-seq results, i.e. the mRNA expression of these genes is significantly upregulated by AZA-treatment in both cell lines (Fig. 7 and Table 5). Also, for 6 genes, the change in DNA methylation levels in the promoter was confirmed by pyrosequencing, even for differential methylated *BDNF* peaks which revealed borderline statistical significance in the MethylCap-seq results (Fig. 8, Figure S3 and Table 5). Note that for *MMP9* the change in promoter methylation was only confirmed in one cell line (ZFTMA). Although *IKBKE* was found to be differentially expressed between both treatments – both with RNA-seq and qPCR – the methylation differences found with MethylCap-seq could not be validated with CpG pyrosequencing (see Discussion).

## Discussion

The methylomes of two zebra finch cell lines were profiled by MethylCap-seq. Both G266 (male, diploid) and ZFTMA (female, tetraploid) cell lines were obtained from spontaneous, non-neuronal tumors and provide an efficient alternative for whole-animal manipulations. As this study is the first to use MethylCap-seq to provide a genome-wide DNA methylation profile of DNA originating from zebra finch samples, we first evaluated the effectiveness of this technique on an untreated ZFTMA cell line. By comparing the MethylCap-seq data with RRBS CpG methylation degrees, MethylCap-seq indeed proved to be an appropriate methodology for genome-wide mapping of DNA methylation in zebra finches. The fact that CpG pyrosequencing validated promoter methylation changes in 6 out of the 7 selected genes further supports our conclusion regarding MethylCap-seq as a suitable methodology in zebra finches, though it only provides a semi-quantitative indication of the degree of methylation.

Based on MethylCap-seq data of AZA-treated samples and controls (solvent, DMSO), an in-house peak calling method identified 719,917 Methylation Peaks of which 30,700 showed significantly less methylation after AZA-treatment. Not unexpectedly, these down-methylated Methylation Peaks were significantly enriched for exonic and promoter regions. Complemented with additional RNA-seq data, we subsequently identified 357 genes featured by expression under putative DNA methylation control, including *ANK1* (Supplementary Table S2). Interestingly, two independent studies recently demonstrated hypermethylation of *ANK1* in the brain of Alzheimer’s patients^41^,^47^. *ANK1* produces ankyrin 1, an essential structural component of the cell outer membranes. Furthermore, this epigenetic alteration appears to occur early on in the disease, making them potential biomarkers. Our results further confirm that *ANK1* expression is regulated by promoter DNA methylation, at least in zebra finch.

Subsequent IPA pathway analysis of the obtained list of genes regulated by DNA methylation showed obvious enrichment for neurological pathways/networks and associations with neurological diseases. Besides neurological diseases and organismal survival, cancer is also in the top three of overrepresented diseases and function categories. This is not surprising, as (i) we are working with cell lines derived from tumor material and (ii) AZA has been shown to have anti-cancer effects by reactivating tumor suppressor genes that are silenced by aberrant DNA methylation^48^. Due to these anti-cancer properties, it has already been used in some clinical trials for leukemia, myelodysplastic syndrome and non-small cell lung carcinoma^49^.

Exon Array validation of RNA-seq results could confirm differential expression for 62.7% of genes, indicating a high level of agreement between RNA-seq and Exon Array results. The possibility of 100% cross-platform agreement is precluded by differences specific to each platform. In contrast to RNA-seq, microarrays have for example the inherent limitation that they can only detect a limited number of genes (in this case 92% of the target genes are detectable by the array) and have a limited dynamic detection range owing to both background and saturation of signals^50^. Moreover, the statistical power and the number of replicates used was different for both platforms, what can further explain the differences in results between platforms.

For a subset of genes putatively regulated by DNA methylation, expression and methylation changes following AZA-treatment were validated using respectively qPCR and CpG pyrosequencing on bisulfite-treated samples. For 6 of the 7 selected genes – *BDNF, NGB, HES1-4, GABRD, ANK1* and *MMP9* – we could validate both promoter methylation and gene expression changes caused by AZA-mediated demethylation. However, whereas the demethylation effect of AZA is very clear in MethylCap-seq data, only modest effects (change of 1–19%) could be observed by pyrosequencing (Supplementary Figure S3). Of special note, though pyrosequencing results demonstrated only moderate methylation changes, subsequent analysis still showed a significant distinction between the DMSO- and AZA-treated samples (Table 5). One possible explanation for this observation is that accumulation of small individual methylation changes in multiple subsequent CpG sequences may culminate in drastic changes in affinity of MethylCap-seq. In contrast, CpG pyrosequencing relies on nucleotide-based sequencing of individual CpG motifs in a stretch of less than 100 bp within a Methylation Peak, so there is always the possibility that there are bigger CpG methylation changes up/downstream of the pyrosequencing amplicon – which could explain why the pyrosequencing results of *IKBKE* were not significant. Alternatively, secondary DNA damage in AZA-treated samples may further reduce the affinity for MBD and overestimate the absolute decrease in DNA methylation levels^51^,^52^. Besides, mixed changes in DNA methylation and hydroxymethylation by AZA-treatment can differentially affect MBD2 binding affinity, whereas bisulfite CpG pyrosequencing can not discriminate between both epigenetic modifications^53^,^54^.

Nevertheless, the magnitude of DNA methylation changes following AZA-treatment is similar to effects reported in gene promoter-specific pyrosequencing assays performed in cancer (patient) samples (human, mouse)^55^.

As already noted in the introduction, songbirds – and zebra finches in particular – are intensively studied by neuroscientists. Dynamic CpG (de)methylation and subsequent gene expression changes play an important role in neuronal functions like learning and memory formation, highlighting the relevance of this study. There are however a couple of important concerns that limit straightforward extrapolation of here reported results to the brain.

1. A first note worth considering is that used cell lines do not originate from brain tissue but from spontaneous, non-neuronal, tumors. However, by comparing the expression profile of these cell lines with the expression profile of the zebra finch auditory lobule, Balakrishnan *et al*.^11^ demonstrated that many neurobiologically relevant genes are expressed in both G266 and ZFTMA cell lines. Moreover, additional overlap with microarray data of Drnevich *et al*. (Songbird Neurogenomics (SoNG) Initiative (SoNG 20 K microarray), 488 songbird brain samples^56^), showed that 104 of the found 357 epigenetically regulated genes were significantly differentially expressed (p < 2.6E-06) in the zebra finch microarray contrasts. This number increased to 134 out of 357 when a less stringent p-value of 0.001 was used to call a gene differentially expressed in the microarray data. A list of these genes is added in the Supplementary Tables (S5). Finally, the fact that ‘neurological diseases’ is the most overrepresented disease category in the pathway analysis together with the plethora of genes associated with brain function in our epigenetic analysis further illustrates the utility of these cell lines to study neurobiologically relevant genes.
2. A subset of tissue-related genes may need tissue-specific transcription factors to be expressed. When these transcription factors are not present in the cell lines, these genes will not be expressed, even if the promoter is demethylated. As a result, our list of 357 genes under putative DNA methylation control could be incomplete for the brain. Therefore, a list of genes with changes in promoter methylation, independent of a change in expression, is also included as Supplementary Table S1.
3. Finally, as G266 and ZFTMA are immortalized cell lines, it is possible that some of the found epigenetically regulated genes are involved in tumorgenesis, particularly when taking into account that (i) DNA methylation deregulation is common in cancer^57^ and (ii) AZA-treatment has been shown to reactivate tumor suppressor genes^48^.
4. The zebra finch reference genome and its annotation are currently still incomplete. This is partially reflected in the rather “low” unique mapping efficiency of the RRBS library. Indeed, besides sequencing quality, also the quality of the reference genome (particularly completeness) and intrinsic features of the studied species (e.g. overall degree of methylation) are anticipated to have a profound impact on mapping percentages. In high quality human RRBS data for example, mapping percentages are approaching 80%^58^. One possibility to increase RRBS mapping efficiencies in this study was to lower the mapping stringency, but as RRBS was particularly used to validate the MethylCap-seq approach rather than to give biological insight as such, this option was not further explored.

We believe that this study can be an important starting point for future epigenetic studies in zebra finch. The genome-wide map of the zebra finch methylome, for both control and AZA-treated samples, allows the identification of sites that are practically always methylated, or for which the methylation status is highly variable in different settings. Furthermore, we provide a list of genes that are putatively regulated by DNA methylation in the promoter region. The next step will be to investigate whether DNA methylation also regulates the expression of these genes *in vivo* and under which circumstances. DNA methylation changes are e.g. known to play an important role during learning processes^59^, brain development^16^ and ageing^60^, and are known to occur in response to (sex) hormone exposure^61^ or environmental cues^62^. Since epigenetic regulation is emerging as an important mechanism in sexual differentiation^63^,^64^ in our future work we will focus on the underlying epigenetic mechanisms of sexual dimorphic brain development during vocal learning in the zebra finch.

In conclusion, using two zebra finch cell lines, we were able to validate the MethylCap-seq methodology in zebra finch samples. To the best of our knowledge, this study provides the first genome-wide DNA methylation map of the zebra finch genome as well as a comprehensive set of genes of which transcription is under putative DNA methylation control. Interestingly, this subset included many neurobiologically relevant genes, further enlightened by the pathway analysis showing obvious enrichment for neurological pathways/networks and associations with neurological diseases. As such, this zebra finch draft methylome may become an attractive data-mining tool for (neuro)epigenetic studies in songbirds.

## Data Submission

The data discussed in this publication have been deposited in NCBI’s Gene Expression Omnibus and are accessible through GEO Series accession numbers GSE61060 and GSE71344 (http://www.ncbi.nlm.nih.gov/geo/query/acc.cgi?acc=GSE61060, http://www.ncbi.nlm.nih.gov/geo/query/acc.cgi?acc=GSE71344).

Next to these files, RRBS data has also been made available in BAM-format, which can be downloaded from the author’s website: http://www.biobix.be/data-2/zebrafinch/.

## Additional Information

**How to cite this article**: Steyaert, S. *et al*. A genome-wide search for eigenetically regulated genes in zebra finch using MethylCap-seq and RNA-seq. *Sci. Rep.* **6**, 20957; doi: 10.1038/srep20957 (2016).

## Supplementary Material

Supplementary Figures

Supplementary Tables

## Figures and Tables

**Table 1:** qPCR primer sequences.

Gene	Forward primer	Reverse primer
*GAPDH*	TCCCATGTTCGTGATGGGTG	GATGGCATGGACAGTGGTCA
*RPS13*	CAGCTCTCCAGAGGAACTCAAGAT	CGCTTGAACACGTTCTTGATGG
*RPL30*	ATGCTTGCCAAGACTGGTGT	GTCAGAGTCACCTGGGTCAA
*BDNF*	CACATCCCGAGTCATGCTAA	ATGTTTGCAGCATCCAGGTA
*NGB*	GGTGATGCTGGTGATTGATG	TCTTCCAGGCAGGACAAGTT
*HES1- 4*	CAGCTGAAGACGCTCATCCT	TTGGAATGCCGGGAGCTATC
*GABRD*	ACCAGAGCTGGAGAGACGAT	GCTTGTCCACAAACCTGCTG
*ANK1*	CAGCGAGATCGTCAACATGC	GTGTGTAATGCAGGGAGGCA
*MMP9*	TTGGTAGCCAAGAGCATGGG	CATCGCTGTTGCCACCATTG
*IKBKE*	ATCGTGGTGGACGTGTTCTC	GCTGCTCTCTGTGGTTTTGC

**Table 2:** CpG pyrosequencing primer sequences.

Gene	Forward primer	Reverse primer (biotinylated)	Sequencing primer
*BDNF*	GTAGAGTTGAGTTGGATAGATGT	^1^ACAATAATTCTACTACTATCCCTTCAA	TGGATAGATGTTTGTATTATATGA
*NGB*	GAGTTAGAATTGATGGGATTAAATAAGG	^1^CCTTACAAAAATAACCAAAAATAACACTTC	GGGTAGTTGGGAGATATA
*HES1-4*	GGTGGGGTTATAAGTTTTTTGAT	^1^CTCCAAAAACATACTACAATTTTCACA	GATAAATTAGAGTGAGGAAAAGAT
*GABRD*	GTAGAAAGTATTTTTGGGTAAAAGTGGTAT	^1^AAGTGGTATTATTTATTTTAGTTAT	TATACACCCAACAACACAAATATATCAA
*ANK1*	TAGTTTATGGGTTAGAAGGATAGGT	^1^ATAAAATCAACACATATTTCCCCTACA	TAGGTGGTGGAGGGT
*MMP9*	AAGTGAGGGTTTATTTTTGAGGTAGTAT	^1^CCCCTAATTTCTCACCATTACTTCCTT	GGTGGGATTTATTTTAGAGT
*IKBKE*	GGGTGAGGGTGTTTGGTATAGT	^1^CCAACCCCTTCTCTTCCTATCA	TGGGAGGGGGTGGTA
Legend: ^1^biotin-labeled			

**Table 3:** Summary of mapping statistics.

a	Cell line	Treatment	Input reads	Uniquely mapped reads	Non-duplicate, uniquely mapped reads	Mapping percentage
MethylCap-seq	G266	DMSO	34,794,421	19,678,423	12,273,057	56.56%
	G266	AZA	36,064,631	15,632,019	5,263,239	43.34%
	ZFTMA	DMSO	29,975,474	13,788,246	9,407,497	46.00%
	ZFTMA	AZA	33,496,076	17,508,214	4,741,451	52.25%
	ZFTMA	Untreated	35,015,944	15,617,024	11,173,357	44.60%
RRBS	ZFTMA	Untreated	73,660,217	20,939,730	*NA*	28.43%
**b**	**Cell line**	**Treatment**	**Input reads**	**Total mapped reads**		**Mapping percentage**
RNA-seq	G266	DMSO	59,068,389	43,896,990		74.31%
	G266	AZA	60,093,607	45,342,201		75.45%
	ZFTMA	DMSO	94,390,205	66,146,096		70.07%
	ZFTMA	AZA	63,714,282	45,870,652		71.99%

(**a**) MethylCap-seq and RRBS. (**b**) RNA-seq.

**Table 4:** Top overrepresented disease and function categories as determined by IPA pathway analysis.

Category	p-value - FDR
Neurological Disease	6,1E-05-6,41E-02
Cancer	3,89E-03-6,41E-02
Organismal Injury and Abnormalities	3,89E-03-6,41E-02
Respiratory Disease	8,51E-03-6,41E-02
Inflammatory Disease	1,03E-02-6,41E-02
Hereditary Disorder	1,03E-02-6,41E-02
Psychological Disorders	1,03E-02-6,41E-02
Skeletal and Muscular Disorders	1,03E-02-6,41E-02
Cell Cycle	1,03E-02-6,41E-02
Amino Acid Metabolism	1,03E-02-6,41E-02
Molecular Transport	1,03E-02-6,41E-02
Small Molecule Biochemistry	1,03E-02-6,41E-02
Gastrointestinal Disease	1,16E-02-6,41E-02
Cardiovascular System Development and Function	1,16E-02-6,41E-02
Organismal Development	1,16E-02-6,41E-02

Categories, corresponding p-values and false discovery rates are shown for the analysis done on the list of 357 AZA-induced upregulated zebra finch genes with at least one significantly down-methylated Methylation Peak in their promoter region.

**Table 5:** Results of qPCR and CpG pyrosequencing validation.

Gene	qPCR		CpG pyrosequencing	
	ZFTMA	G266	ZFTMA	G266
*BDNF*	1.09E-11	6.22E-11	2.03E-4	2.55E-4
*NGB*	3.25E-6	2.12E-8	6.74E-3	3.53E-2
*HES1-4*	3.25E-6	2.12E-8	6.97E-3	8.85E-3
*GABRD*	1.41E-11	3.82E-2	1.09E-3	2.60E-2
*ANK1*	2.03E-5	1.12E-7	6.40E-8	5.03E-08
*MMP9*	7.96E-13	1.71E-14	3.81E-02	2.80E-01
*IKBKE*	1.34E-09	8.06E-08	3.41E-01	5.69E-01

7 genes were selected that showed a significant increase in gene expression (both in RNA-seq and Exon Arrays) and decrease in promoter DNA methylation (MethylCap-seq). qPCR and CpG-pyrosequencing validation experiments were performed comparing expression and methylation values between DMSO- and AZA-treated samples. The obtained gene-specific p-values per cell line clearly show a significant difference in expression for each of the genes for the two treatments. Methylation differences between treatments could be validated by CpG pyrosequencing in all genes except for *IKBKE*. For *MMP9* the change in promoter methylation was only confirmed in the ZFTMA cell line.
