# Corrigendum: A genome-wide search for epigenetically regulated genes in zebra finch using MethylCap-seq and RNA-seq

**DOI:** 10.1038/srep22472

**Published:** 2016-03-17

**Authors:** Sandra Steyaert, Jolien Diddens, Jeroen Galle, Ellen De Meester, Sarah De Keulenaer, Antje Bakker, Nina Sohnius-Wilhelmi, Carolina Frankl-Vilches, Annemie Van der Linden, Wim Van Criekinge, Wim Vanden Berghe, Tim De Meyer

Scientific Reports 6: Article number: 20957; 10.1038/srep20957 published online: 02112016; updated: 03172016

The original version of this Article contained an error in the title of the paper, where the word “epigenetically” was incorrectly given as “eigenetically”. This has now been corrected in the PDF and HTML versions of the Article.

