# Supplementary material for: A genome-wide search for eigenetically regulated genes in zebra finch using MethylCap-seq and RNA-seq
Source: Sci Rep. 2016 Feb 11;6:20957. doi: 10.1038/srep20957 (PMC4750092; doi:10.1038/srep20957)
Supplement: Supplementary Figures [file srep20957-s1.pdf]

A genome-wide search for eigenetically regulated genes in zebra finch using MethylCap-seq and RNA-seq – *Supplementary Figures*

Sandra Steyaert[1*], Jolien Diddens[2], Jeroen Galle[1], Ellen De Meester[1], Sarah De Keulenaer[1], Antje Bakker[3], Nina Sohnius-Wilhelmi[3], Carolina Frankl-Vilches[3], Annemie Van der Linden[4], Wim Van Criekinge[1], Wim Vanden Berghe[2] and Tim De Meyer[1]

[1] Department of Mathematical Modelling, Statistics and Bioinformatics, University of Ghent, Ghent, Belgium.

[2] Laboratory of Protein Chemistry, Proteomics and Epigenetic Signalling (PPES), Department of Biomedical Sciences, University of Antwerp, Antwerp, Belgium

[3] Department of Behavioral Neurobiology, Max Planck Institute for Ornithology, Seewiesen, Germany

[4] Bio-Imaging Lab, Department of Biomedical Sciences, University of Antwerp, Antwerp, Belgium

To whom correspondence should be addressed. Tel: +3292649922; Email: Sandra.Steyaert@Ugent.be

SUPPLEMENTARY FIGURES

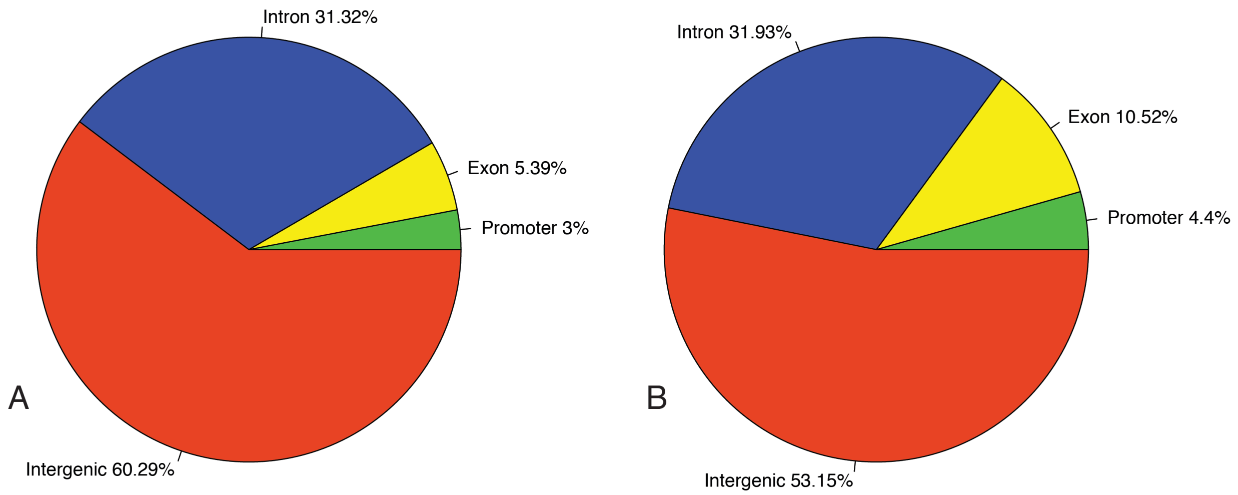

Figure S1: Genomic distribution of the identified Methylation Peaks. (a) Distribution of the full set of 719,917 Methylation Peaks. (b) Distribution of the 30,700 significantly down-methylated Methylation Peaks.

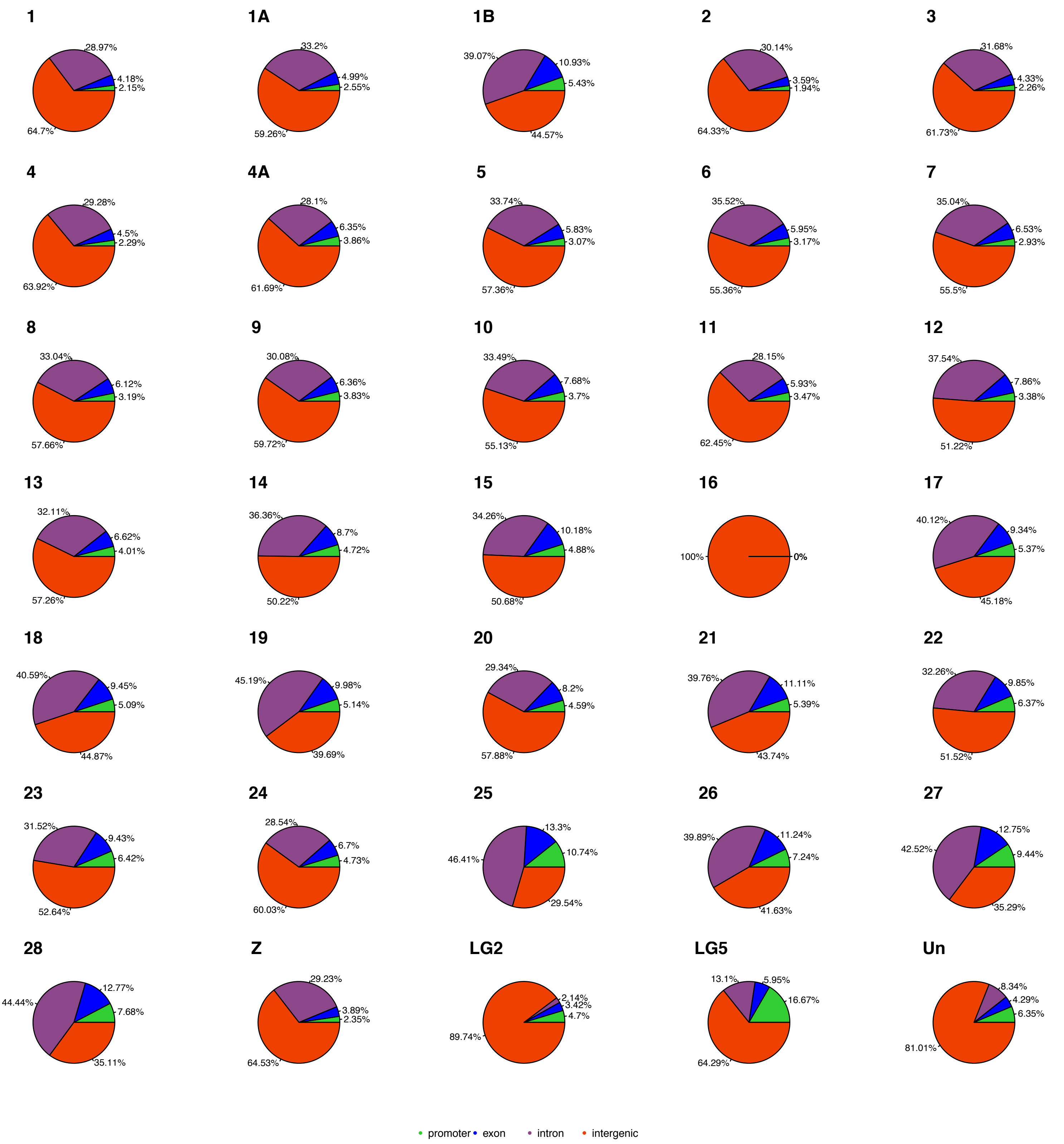

Figure S2(a): Genomic distribution of the 719,917 identified Methylation Peaks per chromosome. Corresponding chromosome names are stated above each piechart. Apart from some exceptions like chromosomes 16, LG2, LG5 and Un, overall, the distribution of the Methylation Peaks follows a general trend.

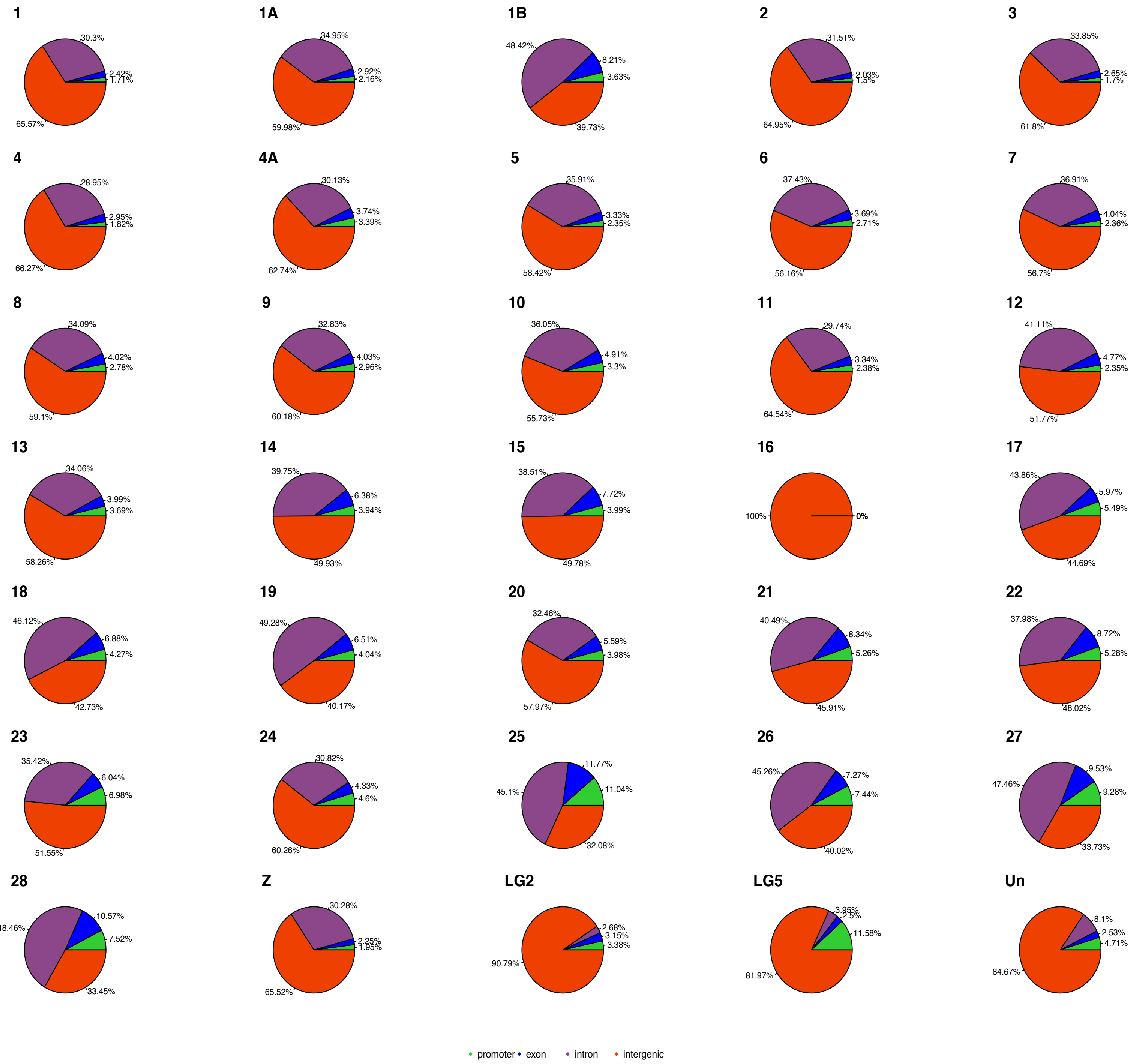

Figure S2(b): Genomic distribution of the summed G266 and ZFTMA coverage of the control (i.e. DMSO-treated) samples per chromosome. Corresponding chromosome names are stated above each piechart. Not unexpectedly, this distribution is very similar to the distribution of Figure S2(a).

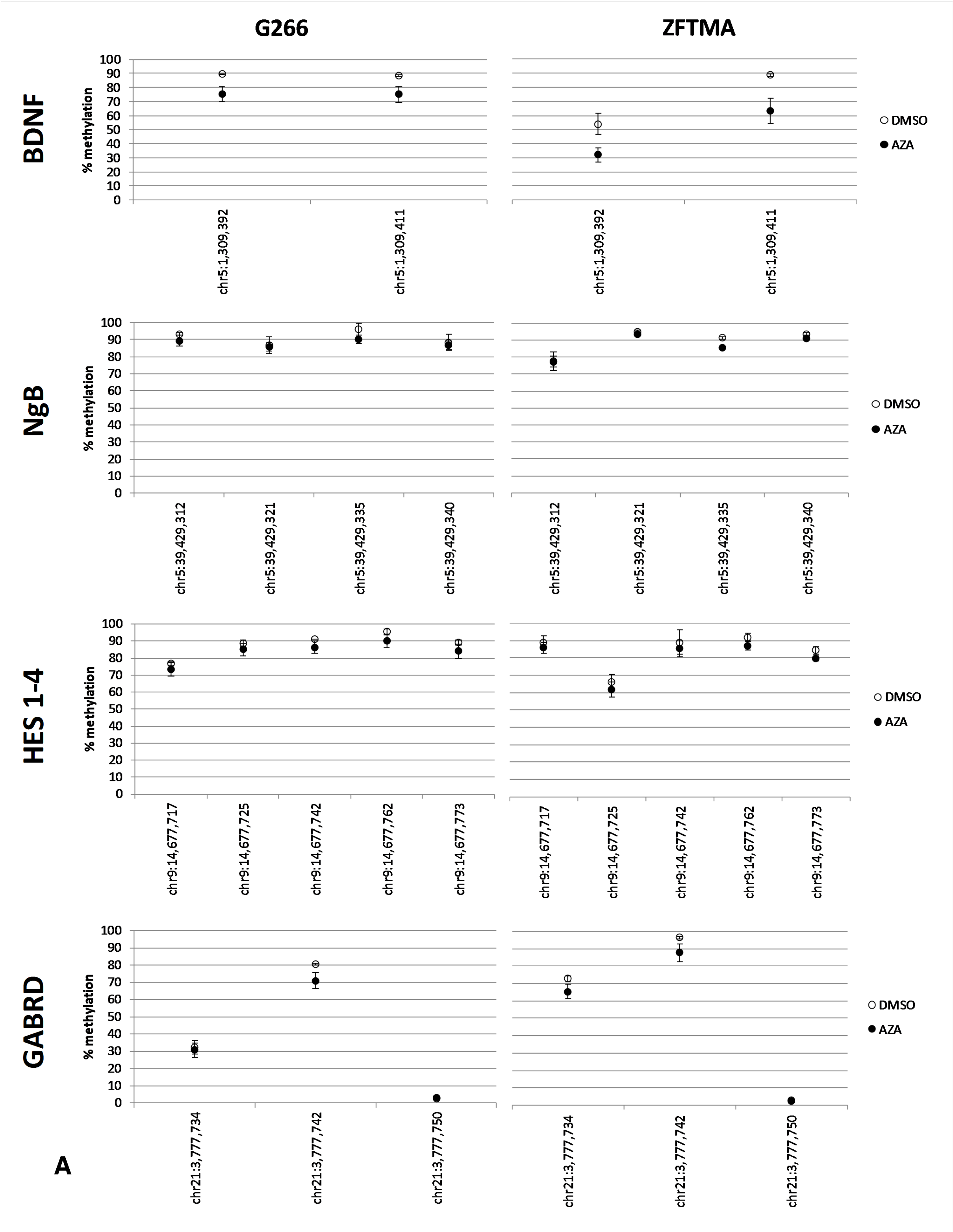
G266
ZFTMA
BDNF
NgB
HES 1-4
GABRD
% methylation
DMSO
AZA
chr5:1,309,392
chr5:1,309,411
chr5:39,429,312
chr5:39,429,321
chr5:39,429,335
chr5:39,429,340
chr9:14,677,717
chr9:14,677,725
chr9:14,677,742
chr9:14,677,762
chr9:14,677,773
chr21:3,777,734
chr21:3,777,742
chr21:3,777,750
A

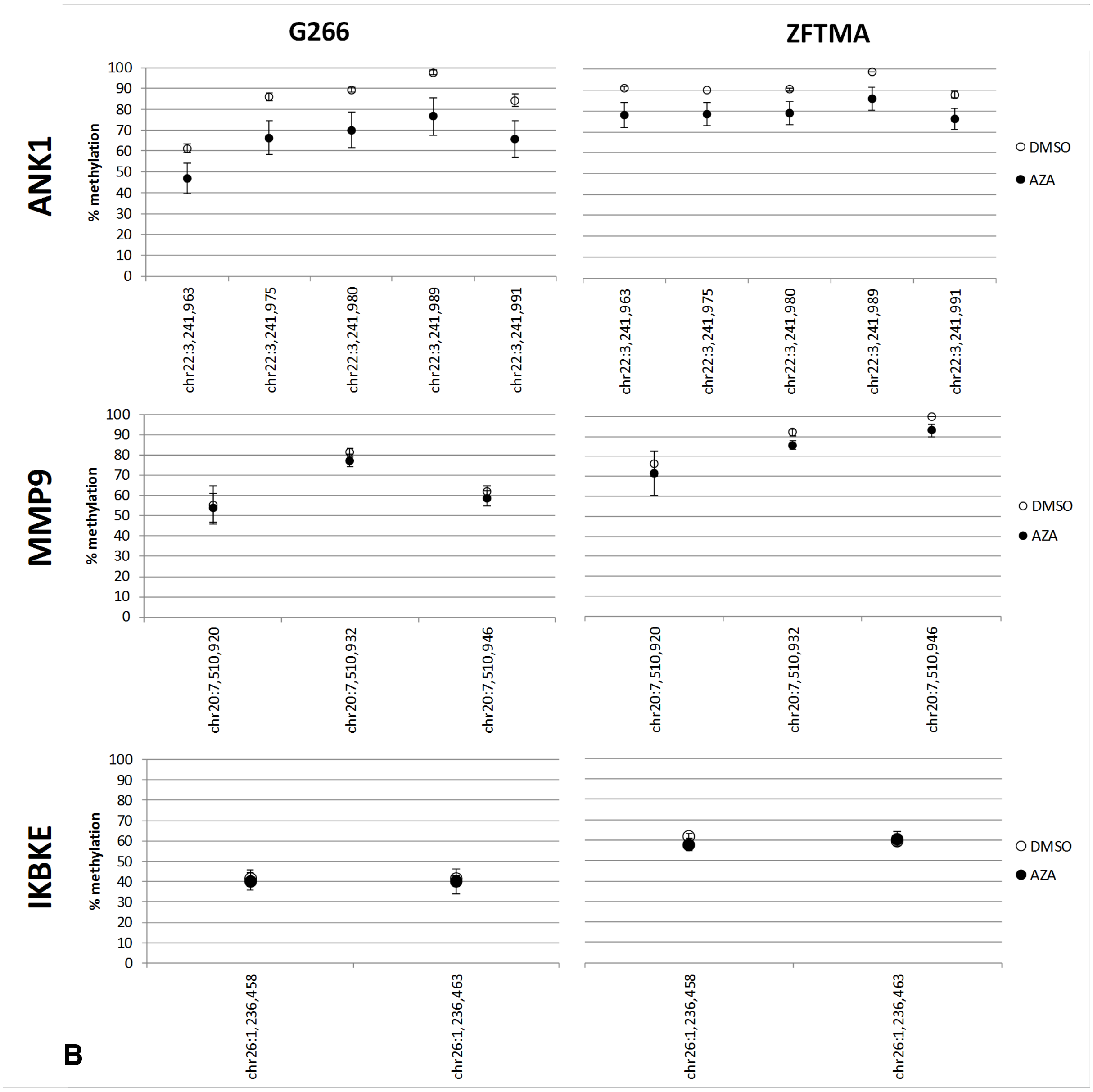

Figure S3: CpG pyrosequencing validation of MethylCap-seq results. This figure shows the changes in DNA methylation of specific CpG sites in the promoters of *BDNF*, *NGB*, *HES1*/4, *GABRD*, *ANK1*, *MMP9* and *IKBKE* after AZA (1 µM) treatment in ZFTMA and G266 cell lines, relative to DMSO solvent control. The shown DNA methylation levels represent the mean of three independent experiments. Only CpG sites that passed quality control were considered. The *NGB* gene was found to contain a SNP in the G266 cell line at position chr5:39,429,316, which was corrected for in the analysis. The methylation changes could be validated for each of the genes, except for *IKBKE* (2-way ANOVA). For *MMP9* the change in promoter methylation was only confirmed in the ZFTMA cell line. *(a) BDNF*, *NGB*, *HES1*/4 and *GABRD. (b) ANK1*, *MMP9* and *IKBKE.*
